# Self-perceived loneliness and depression during the Covid-19 pandemic: a two-wave replication study

**DOI:** 10.14324/111.444/ucloe.000051

**Published:** 2022-11-03

**Authors:** Alessandro Carollo, Andrea Bizzego, Giulio Gabrieli, Keri Ka-Yee Wong, Adrian Raine, Gianluca Esposito

**Affiliations:** 1 Department of Psychology and Cognitive Science, University of Trento, Rovereto, Italy; 2 School of Social Sciences, Nanyang Technological University, Singapore, Singapore; 3 Department of Psychology and Human Development, University College London, London, UK; 4 Departments of Criminology, Psychiatry, and Psychology, University of Pennsylvania, Philadelphia, PA, USA

**Keywords:** Covid-19, depression, lockdown, loneliness, global study, machine learning, SARS-CoV-2

## Abstract

The global Covid-19 pandemic has forced countries to impose strict lockdown restrictions and mandatory stay-at-home orders with varying impacts on individual’s health. Combining a data-driven machine learning paradigm and a statistical approach, our previous paper documented a U-shaped pattern in levels of self-perceived loneliness in both the UK and Greek populations during the first lockdown (17 April to 17 July 2020). The current paper aimed to test the robustness of these results by focusing on data from the first and second lockdown waves in the UK. We tested a) the impact of the chosen model on the identification of the most time-sensitive variable in the period spent in lockdown. Two new machine learning models – namely, support vector regressor (SVR) and multiple linear regressor (MLR) were adopted to identify the most time-sensitive variable in the UK dataset from Wave 1 (n = 435). In the second part of the study, we tested b) whether the pattern of self-perceived loneliness found in the first UK national lockdown was generalisable to the second wave of the UK lockdown (17 October 2020 to 31 January 2021). To do so, data from Wave 2 of the UK lockdown (n = 263) was used to conduct a graphical inspection of the week-by-week distribution of self-perceived loneliness scores. In both SVR and MLR models, depressive symptoms resulted to be the most time-sensitive variable during the lockdown period. Statistical analysis of depressive symptoms by week of lockdown resulted in a U-shaped pattern between weeks 3 and 7 of Wave 1 of the UK national lockdown. Furthermore, although the sample size by week in Wave 2 was too small to have a meaningful statistical insight, a graphical U-shaped distribution between weeks 3 and 9 of lockdown was observed. Consistent with past studies, these preliminary results suggest that self-perceived loneliness and depressive symptoms may be two of the most relevant symptoms to address when imposing lockdown restrictions.

## Introduction

Severe acute respiratory syndrome coronavirus 2 (SARS-CoV-2) is a novel and highly pathogenic coronavirus that originated in bats and was hosted by pangolins before the spillover to humans [1–4]. SARS-CoV-2 disease was first documented in the Hubei province of China in December 2019 and has since rapidly spread throughout the world with the World Health Organisation declaring it a pandemic on 11th March 2020 [5]. As of September 2021, over 224 million people have been infected by Covid-19 and more than 4.6 million deaths have been reported globally [6].

With no available vaccine to prevent Covid-19, many countries were initially forced to adopt lockdown restrictions, which greatly impacted the environments in which people were legally allowed to work, play and socialise – all in the effort to slow down the spread of the invisible virus. Across countries, restrictions varied in period, length and strictness – but all mandates resulted in reduced physical contact between humans in environments that people are used to experiencing. In particular, the UK’s first lockdown announced on 23rd March 2020 imposed a ‘must-stay-home’ order [7], forcing many individuals to renegotiate the home environment as simultaneously also a place of play, learning, rest and socialising. Leaving the house was allowed only once a day for essentials only such as shopping, exercising, medical needs, caring duties and essential travel for work [8]. These restrictions were accompanied by physical distancing measures, which were aimed at reducing the person-to-person transmission of the virus by encouraging the population to stay at least 2 m away from others [9]. Although these policies were effective at reducing the number of new cases and the spread of the airborne virus, individuals had to endure long periods of social isolation, reduced activity in confined indoor spaces, scepticism towards others and little to no contact with others (e.g., friends, parents, siblings, partners), which may have had short- and longer-term impacts on their health.

Considering the impact of social isolation on people’s physical and mental health [10–13], we hypothesised that lockdown measures, specifically lockdown duration (in days), may impact several important aspects of an individual’s daily life. Globally, studies have documented links between restrictions and poorer mental health, such as more post-traumatic stress symptoms, anxiety, depression, insomnia and trust in others [14–18]. Similarly, in a previous data-driven study, we identified that, by using a machine learning model, self-perceived loneliness was most impacted by the time spent in lockdown, over and above other mental health indicators [19]. Further statistical analyses were conducted to assess the variations in participants’ levels of self-perceived loneliness as a function of time spent in lockdown (in weeks). Specifically, participants from the UK who took part in the study during week 6 of the national lockdown reported significantly lower levels of self-perceived loneliness compared to their counterparts who completed the survey during week 3 of the lockdown. Likewise, lower levels of self-perceived loneliness were observed for participants who completed the survey in weeks 4 and 6 of the Greek national lockdown. This pattern of results together with a graphical inspection suggested the existence of a U-shaped distribution in self-perceived loneliness levels by weeks in lockdown in both the UK and Greece. An effect of restrictions on an individual’s perceived loneliness during the first lockdown period was replicated and substantiated by other Covid-19 studies in the literature [20–23].

Building on previous findings, the current study aims to replicate and extend on the previous results. In particular, the current study consists of two parts. In the first part, the work aims to test whether the identification of the most time-sensitive variable by Carollo et al. [19] depended on the chosen machine learning model. To do so, we applied two new machine learning models on the same set of UK data from the first lockdown period to identify the most time-sensitive variable. In this way, we wanted to verify if, when changing the predictive model, new variables with different patterns of time-sensitivity could be identified and studied under a statistical approach. This would provide insight into other time-sensitive variables that might have been overlooked by the previously adopted model – namely, the RandomForest model. In the second part, the study aims to test whether the documented distribution of self-perceived loneliness levels by week in lockdown depended on the specific wave of lockdown. To do so, we graphically analysed self-perceived loneliness distribution by week on data from the second UK national lockdown, with data collected from the UCL–Penn Global COVID Study between 17th October 2020 and 31st January 2021 [24]. The current study provides the opportunity to uncover other aspects that may be significantly influenced by the lockdown restrictions in both the first and second waves of lockdown.

## Methods

### Questionnaire

The current study is based on survey data from the UCL–Penn Global COVID Study, a 12-month study of Covid-19’s impact on mental health in adults conducted between 17th April 2020 and 31st July 2021 [24]. Specifically, this study will use data from Wave 1 collected between 17th April 2020 and 10th July 2020, and data from Wave 2 collected between 17th October 2020 and 31st January 2021. Briefly, the survey was available in eight languages and anyone 18 years and above with access to the survey link through several social media channels (www.GlobalCOVIDStudy.com, email, LinkedIn, WhatsApp, Instagram, Facebook and Reddit) was able to take part in the study. Participants received a randomised presentation of 13 standardised questionnaires assessing mental health including self-perceived loneliness, anxiety, depression, aggression, physical health, social relationships (empathy), living conditions and background variables. For this study, 12 indices derived from the previous questionnaires were included in the analytic sample (see Table 1). As an index of internal reliability, Cronbach’s alpha was computed over the scores based on multiple items.

**Table 1.** Variables that are computed to quantify participants’ mental and physical health and living environment during lockdown

Score	Description	Reference	Domain	Cronbach’s alpha (CI 95%)	Observed range
Mild Activity Difference	Difference between days of mild physical activity post- and pre-Covid-19 lockdown	International Physical Activity Questionnaire – Short Form (IPAQ-SF, 6-items) [25]	Physical Activity	Not applicable	[−7, 6]
Mild Activity Time Difference	Difference between minutes of mild physical activity post- and pre-Covid-19 lockdown	International Physical Activity Questionnaire – Short Form (IPAQ-SF, 6-items) [25]	Physical Activity	Not applicable	[−480, 510]
Moderate Activity Difference	Difference between days of moderate physical activity post- and pre-Covid-19 lockdown	International Physical Activity Questionnaire – Short Form (IPAQ-SF, 6-items) [25]	Physical Activity	Not applicable	[−6, 7]
Sleep Quality	Self-reported sleep quality and quantity, where higher scores reflect better sleep quality	Pittsburgh Sleep Quality Index (2-items) [26], Epworth Sleepiness Scale [27], Subjective and Objective Sleepiness Scale [28]	Sleep Quality	0.73 (0.70–0.77)	[7, 23]
Empathy	Self-reported affective, cognitive, and somatic empathy, where higher scores reflect higher empathy	Cognitive, Affective, Somatic Empathy Scale (CASES, 30-items) [29]	Empathy	0.87 (0.85–0.88)	[29, 60]
Anxiety	Higher scores reflect higher anxiety	General Anxiety Disorder-7 (GAD-7) [30]	Anxiety	0.89 (0.88–0.91)	[0, 20]
Depression	Higher scores reflect higher depression	Patient Health Questionnaire-9 (PHQ-9, 9-items) [31]	Depression	0.87 (0.86–0.89)	[0, 22]
Perceived Loneliness	Higher scores reflect higher perceived loneliness	Loneliness Questionnaire (LQ, 20-items) [32]	Perceived Loneliness	0.94 (0.93–0.95)	[23, 71]
Living Conditions/Environment	Higher scores reflect more chaotic home environments	Chaos, Hubbub, and Order Scale and Health Risk Behaviours (CHAOS, 6-items) [33]	Demographic Information	0.66 (0.62–0.67)	[6, 24]
Beliefs	Perceived effectiveness of government guidelines on social distancing, schools closing, face masks and gloves as protection. Higher scores reflect stronger beliefs	Summed 9-items on Covid-19 beliefs	Worries and Beliefs	0.81 (0.78–0.83)	[19, 45]
Schizotypal Traits	Higher scores reflect more schizotypal traits	Schizotypal Personality Questionnaire–Brief [34]	Social Suspicions and Schizotypal Traits	0.73 (0.70–0.77)	[0, 19]
Reactive-Proactive Aggression	Higher score reflects more aggression	Reactive-Proactive Aggression Questionnaire [35]	Aggression	0.86 (0.84–0.87)	[0, 21]

Cronbach’s alpha was computed on multiple-item scores and it refers to the scores collected during the first wave of lockdown.

This study received ethical approval from the University College London Institute of Education Research Ethics Committee (REC 1331; April 2020).

### Participants

#### Participants from the first wave of lockdown

During the first period of lockdown, a total of 2276 adults from 66 different countries participated in the study. We excluded participants who: i) dissented to take part (n = 32), had incomplete (n = 712) or missing data (n = 165); ii) did not complete the survey within 2 days from the start date (n = 76); iii) filled in the survey from a country that was different from their original country of residence (n = 132). Criterion ii) was applied to exclude possible confounds in the amount of time passed from the start to the end of survey completion. This was a particularly key point in the data processing procedure as we were interested in the effects that the amount of time in lockdown had on people’s mental and physical health. Similarly, criterion iii) was applied to exclude confounds of different types of lockdown restrictions that were adopted by the various countries of the world. All of these participants were excluded from the final analysis.

In contrast to Carollo et al. [19], the current study examined UK participants only. After also excluding the participants who completed the survey after week 9 of lockdown (n = 40), the analytic sample (N = 435) had the following demographic features: female = 345 (79.31%), male = 81 (18.62%), non-binary = 4 (0.92%), prefer not to say = 2 (0.46%), self-identified = 3 (0.69%); age: range = 18–88 years, mean = 37.62, standard deviation (SD) = 13.83 (missing = 1).

#### Participants from the second wave of lockdown

With regard to the second wave of lockdown, 2280 participants completed the survey. The same exclusion criteria described in the section above were applied to Wave 2 data. Thus, 1341 and 140 participants were excluded because they had incomplete and missing data, respectively. Another 206 were excluded because they did not complete the survey within 2 days. Finally, 43 did not fill in the survey from their original country of residence and, therefore, were excluded from the analysis.

To be consistent with the sample used in our previous study, the statistical analysis applied to uncover the pattern of self-perceived loneliness in Wave 2 was conducted uniquely on the UK participants (n = 263). The sample had the following demographic features: female = 216 (82.13%), male = 39 (14.83%), non-binary = 5 (1.90%), prefer not to say = 2 (0.76%), self-identified = 1 (0.38%); age: range = 18–89 years, mean = 38.28, *SD* = 13.74 (missing = 2).

### Data analysis

All the scripts for the data analysis are available at the following link: https://doi.org/10.5522/04/20183858. Prior to data analysis, we computed the variable ‘Weeks in lockdown’ for each participant in both Wave 1 and Wave 2 of the UK national lockdown. The variable ‘Weeks in lockdown’ corresponds to the difference between the date in which the UK adopted lockdown preventive measures (either the beginning of the first or the second lockdown wave) and the survey completion date. This new numerical variable referred to the week of lockdown into which the single participant completed the survey. Table 2 reports the number of participants by week across the first and second waves of the UK national lockdown.

**Table 2.** Number of participants from the UK by week during the first and second period of lockdown

Wave of lockdown	Before week 3	Week 3	Week 4	Week 5	Week 6	Week 7	Week 8	Week 9	After week 9	TOT
Wave 1	0	42	100	80	76	110	23	4	0	435
Wave 2	244	5	2	3	1	0	0	4	4	263

Using data from Waves 1 and 2 of the UCL–Penn Global COVID Study and the same health variables across both time-points, we conducted two sets of analyses to answer our research questions. To test whether the identification of the most time-sensitive variable in Carollo et al. [19] depended on the chosen machine learning model, we used Wave 1 data and we adopted a data-driven machine learning approach. As compared to the RandomForest model adopted in Carollo et al. [19], in the current work we used two different machine learning models to identify the most time-sensitive variable (out of the 12 indices included). The distribution of scores by week of the identified most time-sensitive variable was then examined through a statistical approach with significance tests corrected for multiple comparisons.

To test whether the U-shaped pattern of self-perceived loneliness found in Carollo et al. [19] was unique to Wave 1 of the lockdown, we used Wave 2 data to conduct a graphical inspection of the distribution of scores by week in lockdown.

#### Data-driven and statistical replication of the results in Wave 1

The current paper first adopted a machine learning approach to test whether the identification of the most time-sensitive variable in Carollo et al. [19] was specific to the RandomForest model or whether we would replicate the result using new models – namely, support vector regressor (SVR) [36] and multiple linear regressor (MLR). While RandomForest’s predictions are based on the creation of an ensemble of decision trees from the input variables, SVR is rooted in the derivation of a best-fit hyperplane and MLR on linear relations between variables. Data from 12 variables of interest (outlined in Table 1) were included in the models to predict the independent variable ‘Weeks in lockdown’. The assumption behind this approach was that the independent variable ‘Weeks in lockdown’ would modulate, to a different extent, the scores of the dependent variables included in the dataset. Particularly, the most time-sensitive variable would be strongly modulated by time in lockdown and its scores would systematically co-vary with the variable ‘Weeks in lockdown’. Therefore, the most time-sensitive variable would also be the most informative and important for the model when trying to predict ‘Weeks in lockdown’. Under these assumptions, first, we applied a standardised 10 × 5-fold cross-validation scheme to train the SVR and the MLR on 75% of the data. Once the models were established, we then applied them to the remaining 25% of data, the ‘testing set’ data. The cross-validation and the train-test split procedures are common practice in machine learning as they help to control the model’s overfitting by evaluating the model’s performances on unseen data [37]. Overall, the models’ accuracy was assessed by comparing real and predicted values. In particular, the models’ performances were evaluated by mean squared error (MSE), which consists of the average squared difference between predicted and real values. Thus, a lower MSE value corresponds to a higher overlap between the real and predicted data. For every training iteration, the variables were ranked by their absolute coefficient value to reflect their influence on the model’s built. On all the training importance rankings, we computed a Borda count to determine the most important and informative variable for the model’s prediction of the weeks in lockdown. The Borda count is a method to derive a single list summarising the information coming from a set of lists [38]. For the SVR model, by comparing the several training evaluation iterations, we derived the optimal hyper-parameter *C*. In SVR, the parameter *C* is a cost regularisation parameter which determines the trade-off cost between minimising the training error and minimising model complexity [39]. The resulting optimised *C* parameter was equal to the value of 0.01, and it was implemented in the final model. The final models (i.e., SVR with *C* parameter set at 0.01 and the MLR) were then trained by using all the data from the training set and their performances were evaluated on the testing set data.

Next, focusing on the most time-sensitive variable identified with the SVR and MLR models, we applied a multipair Kruskal–Wallis test to assess whether the variable scores changed over the lockdown period. The Kruskal–Wallis test represents the non-parametric counterpart of analysis of variance. The Kruskal–Wallis test was chosen because it requires fewer assumptions to be conducted as compared to its parametric counterpart [40]. In this study, scores from participants belonging to weeks 3 (since at the beginning of the data collection, the UK lockdown was already started) to 7 were compared. As the study had a cross-sectional design across waves of lockdown, participants were grouped by the ‘Week in lockdown’ variable. ‘Week in lockdown’ groups were compared in terms of scores reported for the identified most time-sensitive variable. In this way, a significant result in the multipair Kruskal–Wallis test would indicate that levels of the identified variable significantly differed by ‘Weeks in lockdown’ for at least two groups of weeks. If the multipair Kruskal–Wallis test suggested the existence of significant weekly variations, we conducted multiple pairwise Kruskal–Wallis tests with a Bonferroni correction to compare week 7 scores to other weeks. Eta (*η*^2^)-squared was computed to estimate the magnitude of significant results [41,42].

#### Graphical replication of the results in Wave 2

To test whether the distribution of weekly self-perceived loneliness levels was unique to Wave 1 of lockdown, a graphical qualitative inspection was conducted on Wave 2 data. Again, participant’s self-perceived loneliness scores were clustered by week of lockdown and the distribution of scores from weeks 3 to 9 was inspected with boxplots. It is worth noting that, considering the limited sample size that was available for Wave 2 from weeks 3 to 9, no statistically meaningful insight could be derived from the comparisons of groups, so the second part of the study can only have a qualitative and descriptive significance, and must be considered as a preliminary approach.

## Results

### Replication of the results in Wave 1

**Figure 1:**
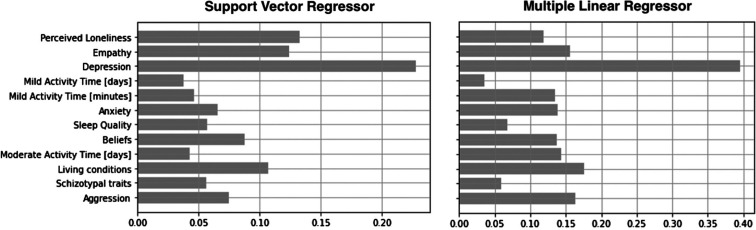
Normalised average importance of the selected variables when training a SVR model (on the left) and a MLR (on the right) on data from the first lockdown period. The importance of the variables was derived from the trained predictive models as the absolute value of the variables’ weights or coefficients for the SVR and MLR, respectively.

MSEs for the SVR performances were 2.04 and 2.29 for the training and test data, respectively. For the MLR, MSEs were 1.97 and 2.39 for the training and test data, respectively. While both models’ performances on the training set are slightly worse than in Carollo et al. [19], the performances on the test are in line with the previous paper. Furthermore, depression scores were found to be the most informative for both the SVR and MLR’s training, above and beyond the other variables in the models (see Fig. 1).

**Figure 2:**
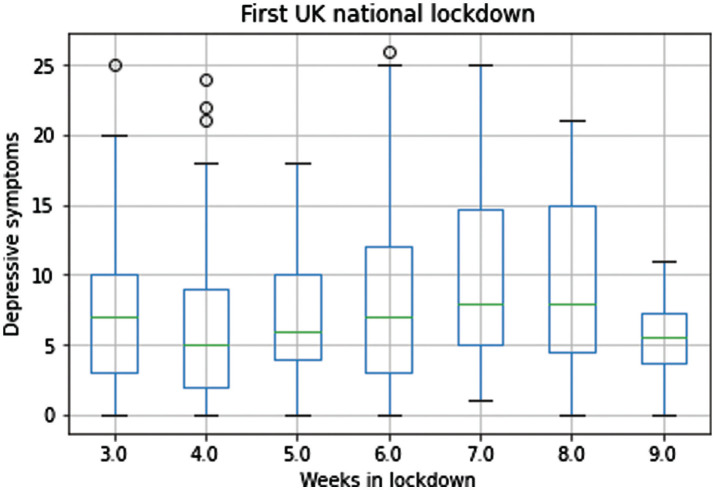
Symptoms of Depression reported by week during the first UK national lockdown.

A closer look at boxplots representing depressive symptoms divided by week in lockdown suggests that, from weeks 3 to 7, the median score decreased in the first period (week 3 to week 4) and then increased again (from week 4 to week 7; see Fig. 2). A decrease followed by an increase in scores suggests a U-shaped pattern for depressive symptoms in the first wave of the UK lockdown.

A Kruskal–Wallis test confirmed that at least 1 week (in the period from the 3rd to the 7th week of lockdown) differed significantly from the others in terms of depressive symptoms (H = 22.03, *P* < 0.001, *η*^2^ = 0.042). Specifically, symptoms between week 4 and week 7 (H = 22.52, *P* < 0.001, *η*^2^ = 0.050), and between week 5 and week 7 (H = 9.69, *P* = 0.002, *η*^2^ = 0.020) were statistically different. Conversely, the comparisons between week 3 to week 7 (H = 4.64, *P* = 0.031), and week 6 to week 7 (H = 4.02, *P* = 0.045) were not significant after applying the Bonferroni bias-correction.

### Qualitative replication of the results in Wave 2

**Figure 3:**
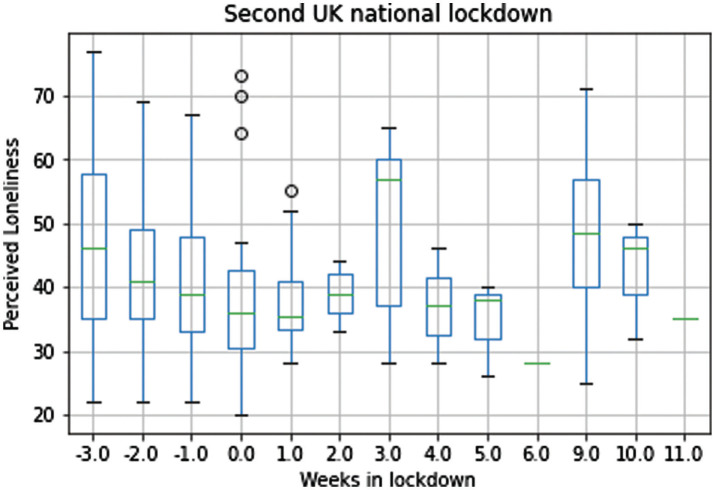
Reports of Perceived Loneliness by week during the second UK national lockdown.

A graphical inspection of boxplots with self-perceived loneliness scores divided by week suggests that, between weeks 3 and 9 of Wave 2 of the UK national lockdown, another U-shaped pattern could be reported. Specifically, participants who took part at the study during the 4th and 5th weeks of lockdown reported lower levels of self-perceived loneliness than did participants in the survey during week 3. Although there were not enough participants for weeks 6, 7 and 8, self-perceived loneliness scores during week 9 were reportedly higher again (see Fig. 3).

## Discussion

This study applying a machine learning approach alongside a statistical approach to data from Waves 1 (17 April to 31 July 2020) and 2 (17 October 2020 to 31 January 2021) of the UCL–Penn Global COVID Study [24] identifies the mental health variable(s) most influential in predicting the UK lockdown duration, and how the variable varies by week. This gives an indication of how people were fairing when confined in the limited, often shared, space in which they have to work, learn, play and rest. With the aim of replicating and extending the results from our previous paper [19], we applied a support vector regressor (SVR) model and a multiple linear regressor (MLR) model instead of a RandomForest model to predict participants’ weeks in lockdown. Based on the variables’ importance ranking, depressive symptoms, over and above the other 11 health indices, were the most important variable for both the SVR and MLR models when determining the model best-fit to the data and were the best at predicting lockdown duration in weeks. Depressive symptoms were therefore identified by both the SVR and MLR models as the most time-sensitive variable in the dataset. As the focus of the study was not to assess the variables’ predictive capability per se, it is worth noting that the low model performance did not affect the reliability of the variable importance ranking and, therefore, the identification of the most time-sensitive variable in the dataset [19]. Specifically, depressive symptoms reported across the 9 lockdown weeks resulted in a U-shaped pattern where symptoms were lowest during weeks 4 and 5 compared to week 7.

Variation in the population’s depressive symptoms during lockdown has been reported by past studies as depressive symptoms have been a key mental health issue during the Covid-19 pandemic [43–46]. Specifically, Ammar et al. [47] compared the scores pre- and post-lockdown in symptoms of depression and found higher depressive symptoms as a result of home confinement. Notably, this study relied on self-report ratings of depression from participants internationally (e.g., Asia, Europe and Africa), thus further substantiating the reliability of our finding. This is not surprising, given that social isolation is a common precursor of poorer mental and physical health [48], with increased risk for depression [49–51]. In another study by Delmastro et al. [52] of the lockdown in Italy, people living alone, or not being allowed to leave the house to go to work, tended to have higher depressive symptoms. Like self-perceived loneliness, symptoms of depression have varied during the first UK lockdown. Self-report data from the United States during their first 3 months of lockdown also showed that self-perceived loneliness was positively correlated with depression and suicide ideation at various time-points [53]. In fact, during the Covid-19 pandemic, self-perceived loneliness – a discrepancy between desired and perceived social connection – seemed to be one of the most important risk factors for depression (and anxiety) [54], and social trust [18]. Specifically, higher perceived social support during lockdown – in other words, lower self-perceived loneliness – was associated with lower depressive symptoms [55]. After such periods, instead, self-perceived loneliness appeared to act as a moderator between stress and depression [56].

While the limited sample size by week in Wave 2 data did not allow the statistical approach adopted in Carollo et al. [19] to be used, a graphical U-shaped pattern of self-perceived levels of loneliness seems to emerge again across the lockdown weeks. Again, qualitatively, the self-perceived levels of loneliness were low during weeks 4 and 5, and highest during the 3rd and 9th weeks of the lockdown period. These results have to be considered only as a qualitative and preliminary insight, as the sample size collected for the weeks of interest did not allow any meaningful statistical inference to be made. In fact, graphical disparities among scores might be mere random variation and they might not reflect real differences. Nonetheless, our study findings suggest that local and nationwide initiatives to help reduce self-perceived loneliness and increase solidarity and community cohesion may be helpful at improving people’s mental health during lockdowns.

In conclusion, both self-perceived loneliness and depressive symptoms appear to follow U-shaped curves across periods of lockdown (although no statistical test was computed over scores of self-perceived loneliness by week in the second wave of the UK lockdown). Knowing the unfolding of these trajectories might be helpful for conveying adequate support to the population in lockdown with the right timing. People might also be made aware of the possible fluctuations in self-perceived loneliness and depressive symptoms throughout the lockdown period. Overall, this knowledge can help manage expectations in populations and support systems to ensure that resources are allocated effectively, especially in future lockdown environments. Of course, ‘why’ both perceived levels of loneliness and depression follow U-shaped patterns will necessarily involve the examination of individual-level characteristics (e.g., age, gender), or other variables, that were not assessed and explored in the current study. For the same aim, a longitudinal investigation – opposed to the cross-sectional design of the current study – could also provide useful results. Furthermore, to fully pursue the replication aims of the current study, it would be useful to apply the same machine learning and statistical approach across different data sources. As we did not find any dataset similar enough to the one we adopted, the results from the current paper can only be considered as preliminary. Although these are limitations, the present study also has some clear strengths. First of all, a wide range of mental and physical variables could be studied in a data-driven fashion thanks to the adopted machine learning approach. In this way, we were able to identify and, in a second phase, statistically characterise the index that varied the most accordingly to the time spent in lockdown. Moreover, given the differences across lockdown restrictions, cross-cultural comparisons of the impacts of Covid-19 on populations are challenging. Thus, a strength of the current study is to focus just on the UK. Generally, the study highlighted the importance of considering the potential weekly variation in mental health across a wide range of variables and the variation that may exist across individuals and countries with different lockdown restrictions.

## Data Availability

The datasets generated during and/or analysed during the current study are available in the repository: https://doi.org/10.5522/04/20183858.
